# Nomograms and scoring system for forecasting overall and cancer‐specific survival of patients with prostate cancer

**DOI:** 10.1002/cam4.5137

**Published:** 2022-08-22

**Authors:** Yuan Zhou, Changming Lin, Lian Zhu, Rentao Zhang, Lei Cheng, Yuanyuan Chang

**Affiliations:** ^1^ Department of Urology Surgery The People's Hospital of Xuancheng City Xuancheng China; ^2^ Wannan Medical College Wuhu China; ^3^ Department of Urology Surgery The Fourth Affiliated Hospital of AnHui Medical University Hefei China; ^4^ Department of Pulmonary Medicine Shanghai Chest Hospital, Shanghai Jiao Tong University Shanghai China

**Keywords:** nomogram, prognosis, prostate cancer, SEER, survival

## Abstract

**Background:**

Estimated life expectancy is one of the most important factors in determining treatment options for prostate cancer (PCa) patients. However, clinicians have few effective prognostic tools to individually assess survival in patients with PCa.

**Methods:**

We screened 283,252 patients diagnosed with PCa from the Surveillance, Epidemiology, and End Results (SEER) database between 2004 and 2015, and randomly divided them into the training and validation groups. We used univariate and multivariate Cox analyses to identify independent prognostic factors and further established nomograms to predict 1‐, 3‐, 5‐, and 10‐year overall survival (OS) and cancer‐specific survival (CSS) for PCa patients. The prediction performance of nomograms was tested and externally validated by Concordance index (C‐index) and receiver operating characteristic (ROC) curve. Calibration curve and decision curve analysis (DCA) were used for internal validation. We further developed PCa prognostic scoring system based on the impact of available variables on survival.

**Results:**

The variables age, race, marital status, TNM stage, surgery method, radiotherapy, chemotherapy, PSA value, and Gleason score identified as independent prognostic factors were included in the survival nomograms. The results of training (C‐index: OS = 0.776, CSS = 0.889; AUC value: OS = 0.772–0.802, CSS = 0.892–0.936) and external validation (C‐index: OS = 0.759, CSS = 0.875) indicated our nomograms had good performance in predicting 1‐, 3‐, 5‐, and 10‐year OS and CSS prediction. Internal validation using the calibration curves and DCA curves demonstrated the effectiveness of the prediction models. The prognostic scoring system was more effective than the AJCC staging system in predicting the survival of PCa patients, especially for OS.

**Conclusion:**

The prognostic nomograms and prognostic scoring system have favorable performance in predicting OS and CSS of PCa patients. These individualized survival prediction tools may contribute to clinical decisions.

## Graphical abstract

Based on a large data set of prostate cancer patients, we developed and valdated prognostic nomograms and demonstrated their favourable ability in predicting 1‐, 3‐, 5‐, and 10‐year overall and cancer‐specific survival for prostate cancer patients. We further proposed the prognostic scoring system for the first time to more intuitively present the influence of clinical pathological factors on overall and cancer‐specific survival of prostate cancer. The proposed survival model of prostate cancer can help clinicians select individualized and effective treatment.

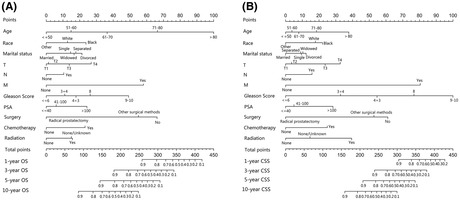

## 1 INTRODUCTION

As a global threat to men's health, prostate cancer (PCa) has attracted wide attention. According to statistics released in 2020, there were approximately 191,930 new cases of PCa in the United States, accounting for 21.5 percent of all male malignancies.^1^ Among all stages combined, PCa has the highest 5‐year survival rate of 98 percent, higher than the 67 percent average for all malignancies.^2^ Radical prostatectomy is the preferred treatment for localized PCa, and the surgical indications have extended to some patients with locally advanced disease in recent years.^3^, ^4^, ^5^ Estimated life expectancy is one of the most important factors in determining treatment options for PCa patients.^6^ Current consensus guidelines recommend that radical prostatectomy is the first choice of treatment for PCa patients in low‐ and intermediate‐risk localized groups with a life expectancy of more than 10 years and high‐risk localized groups with a life expectancy of more than 5 years.^7^ Radiotherapy combined with androgen deprivation therapy is the preferred treatment for localized PCa patients with a life expectancy of less than 5 years and for all advanced PCa patients.^7^ Life expectancy greatly influences the treatment choice and intensity of PCa patients. Some scholars had proposed that the survival time of patients can be evaluated according to their age and gait speed.^8^ Some clinicians established charlson comorbidity index to predict the survival time of PCa patients based on race, sex, comorbidity, and social life expectancy.^9^ However, these prognostic systems cannot personalize the patient's survival in combination with clinicopathological factors such as age, marital status, pathological grade, tumor stage, surgical, and chemoradiotherapy methods.

Nomogram, a statistical tool that can plot multiple independent risk factors into an intuitive graph, has been used extensively in recent years to predict survival time for patients with various cancers.^10^, ^11^, ^12^ Thus, the purpose of this study is to establish a nomogram prognostic tool to accurately and individually estimate overall survival (OS) and cancer‐specific survival (CSS) for PCa patients to help clinicians select treatment options and intensity.

## 2 MATERIALS AND METHODS

### 2.1 Data source

The clinicopathological data of all patients in this study were obtained from the SEER database (Surveillance, Epidemiology and End Results) database [SEER18 Regs Custom Data, based on the 2018 submission]. The SEER database, supported by the National Cancer Institute, is the largest registry of cancer patients in the United States.^13^ The SEER database is public and identifiable, so informed consent of patients and ethics committee permission is not required for this study.

### 2.2 Patients and clinicopathologic factors

We set inclusion criteria for patients in this study: (a) Patient pathologically diagnosed with adenocarcinoma of PCa between 2004 and 2015; (b) Patients had no previous history of other cancers out of PCa; (c) The clinicopathological information of included patients were available from the hospital, including race, age at diagnosis, marital status, TNM stage, surgery method, radiotherapy, chemotherapy, prostate‐specific antigen (PSA) value, Gleason score, OS state, CSS state, and survival data. We excluded patients with missing information on above mentioned. (d) We excluded patients with scarce Gleason scores, such as Gleason 1 + 4, 2 + 5 and 3 + 1.

### 2.3 Statistical analysis

We randomly divide PCa patients diagnosed in year of 2004, 2010, and 2012 as the validation cohort, and the remaining patients were used as the training cohort to establish nomogram models. We identified independent prognostic factors with *p* values <0.05 in the multivariate Cox analyses. Effective prognostic factors were further used to develop nomograms to predict 1‐, 3‐, 5‐, and 10‐year OS and CSS rates in patients with PCa. Receiver operating characteristic (ROC) curves and nomogram were used to establish the survival model. Area under curve (AUC) and C‐index were computed to quantify the predictive ability of the survival models. Then we validated the accuracy of the nomogram models externally. Internally validation was performed by decision curve analysis (DCA) to further evaluate the practicability of the nomogram models for clinical decision‐making. We further established prognostic score for OS and CSS of PCa patients based on the coefficient in Cox models for each variable. The prognostic score was ranged from 0–100 and was used to divide the prognosis of PCa patients into four grades. We used Kaplan–Meier curves and Akaike information criterion (AIC) to compare OS and CSS in PCa patients between the prognostic grading system and AJCC staging system.

## 3 RESULTS

### 3.1 Patient clinicopathological data

**FIGURE 1:**
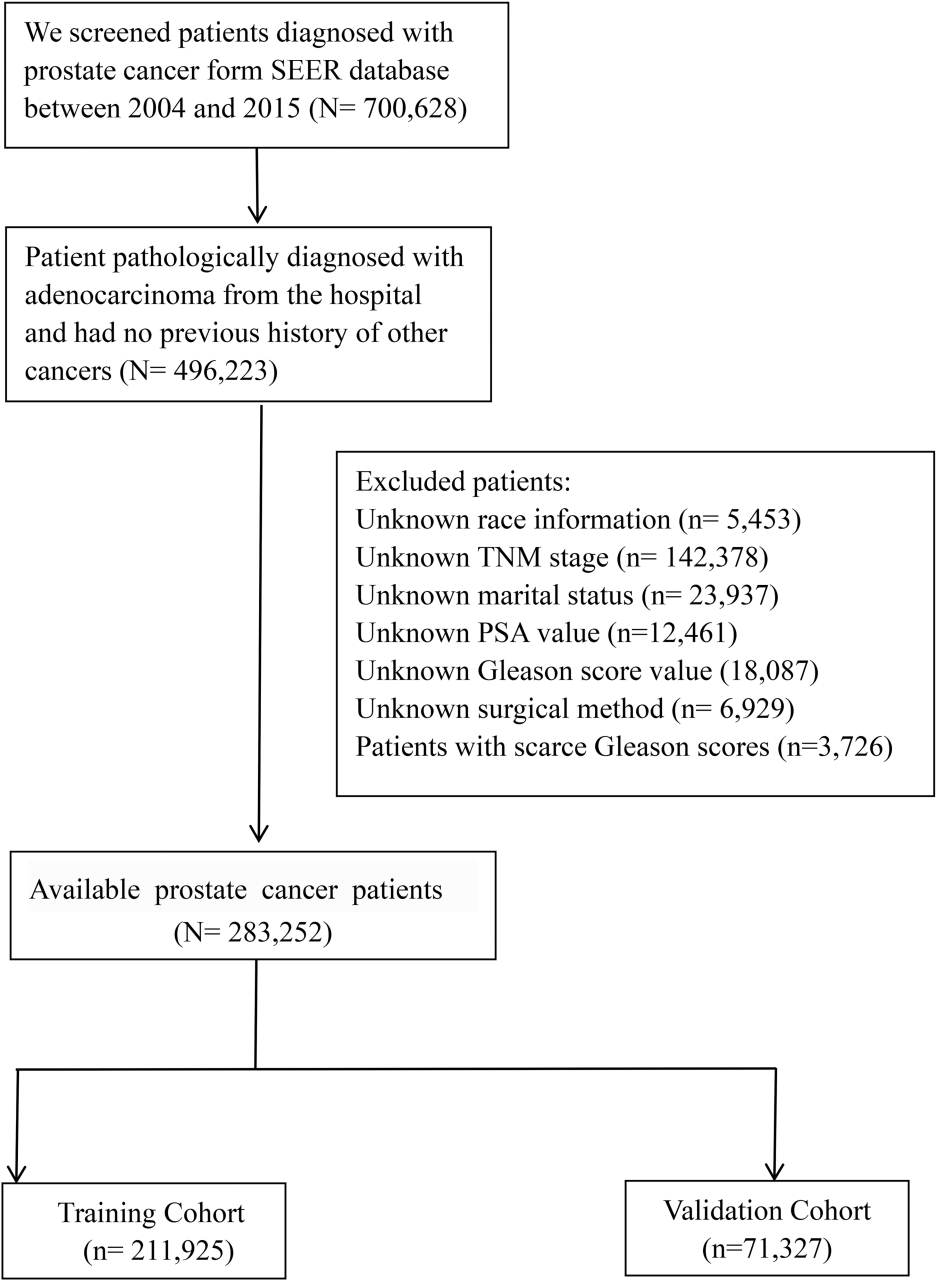
Flow chart of prostate cancer patients screening form SEER database.

According to the selection criteria, 283,252 patients with PCa were screened in this study, among which patients were divided into training cohort and validation cohort (Figure 1). The 1‐, 3 ‐, 5 ‐, and 10‐year OS and CSS rates in the training cohort were similar to those in the validation cohort (OS: training cohort vs. validation cohort, 98.6%, 94.7%, 90.3%, and 76.6% vs. 98.6%, 94.7%, 90.2%, and 75.4%, respectively; CSS: training cohort vs. validation cohort, 99.4%, 98.0%, 96.9%, and 93.6% vs. 99.4%, 98.1%, 96.8%, and 93.3%, respectively). We listed the baseline characteristics of PCa patients in Table 1. We compared the distribution of invasion factors in PCa, and found that PCa patients with higher Gleason grade tended to have higher PSA and T staging, and were prone to have regional lymph nodes and distant metastases (Table S1).

**TABLE 1:** Baseline characteristics of prostate cancer patients (*n* = 283,252, 2004–2015), among with patients diagnosed in year of 2004, 2010, and 2012 were divided into the validation cohort, and the remaining patients were divided into the training cohort

Patient characteristics	Training cohort (*n* = 211,925)		Validation cohort (*n* = 71,327)	
	No. of patients	%	No. of patients	%
Age				
<=50	9939	4.7	3533	5.0
51–60	58,463	27.6	19,554	27.4
61–70	91,325	43.1	30,807	43.2
71–80	44,491	21.0	14,835	20.8
>80	7707	3.6	2598	3.6
Race				
White	166,614	78.6	56,156	78.7
Black	34,040	16.1	11,394	16.0
Other	11,271	5.3	3777	5.3
Marital status				
Married	161,240	76.1	54,406	76.3
Divorced	16,395	7.7	5546	7.8
Separated	2012	1.0	672	0.9
Widowed	8437	4.0	2862	4.0
Single	23,841	11.2	7841	11.0
T (tumor invasion)				
T1	94,651	44.7	31,561	44.2
T2	86,530	40.8	29,808	41.8
T3	26,778	12.6	8631	12.1
T4	3966	1.9	1327	1.9
*N* (regional lymph node)				
No	206,111	97.3	69,563	97.5
Yes	5814	2.7	1764	2.5
M (metastasis)				
No	206,825	97.6	69,731	97.8
Yes	5100	2.4	1596	2.2
Surgery				
No	106,035	50.0	35,438	49.7
Radical prostatectomy	94,487	44.6	32,196	45.1
Other surgical methods	11,403	5.4	3693	5.2
Radiation				
Yes	79,060	37.3	26,820	37.6
No	1237	0.6	466	0.7
None/Unknown	131,628	62.1	44,041	61.7
Chemotherapy				
None/Unknown	210,657	99.4	70,980	99.5
Yes	1268	0.6	347	0.5
Gleason score				
<=6	90,896	42.9	33,340	46.7
3 + 4	61,970	29.2	19,239	27.0
4 + 3	25,557	12.1	8093	11.4
8 (3 + 5/4 + 4/5 + 3)	18,272	8.6	6008	8.4
9–10 (4 + 5 /5 + 4/5 + 5)	15,230	7.2	4647	6.5
PSA value				
<=40	27,724	13.1	9206	12.9
41–100	130,359	61.5	43,860	61.5
>100	53,842	25.4	18,261	25.6
Death status				
Alive	180,916	85.4	58,945	82.6
Death	31,009	14.6	12,382	17.4

*Note*: The training cohort was used to establish nomograms and internal validation, and the validation cohort was used to externally verify the prediction accuracy of nomograms.

### 3.2 Independent predictors for patients

The results of univariate and multivariate analyses for OS and CSS in the training cohort were presented in Table 2. Variables with *p* values <0.05 in both Cox analyses were considered as independent prognostic factors. The multivariate Cox analyses indicated older age (≥80 vs. ≤50 years old, HR = 7.23; 95%CI = 6.57–7.95; *p* < 0.001) and M1 stage (M1 vs. M0, HR = 3.04; 95%CI = 2.90–3.19; *p* < 0.001) were the risk factors most associated with overall death. With regard to CSS, higher Gleason score grade (grade 5 [Gleason 9–10] vs. grade 1 [Gleason≥6], HR = 10.23; 95%CI = 9.47–11.06; *p* < 0.001) and M1 classification (M1 vs. M0, HR = 5.54; 95%CI = 5.18–5.92; *p* < 0.001) were the highest risk factors for cancer‐specific death (Table 2).

**TABLE 2:** Univariate and multivariate COX analyses for overall survival (OS) and cancer‐specific survival (CSS) of prostate cancer patients in the training cohort

Patient characteristics	OS			CSS		
	Univariate	Multivariable		Univariate	Multivariable	
	*p* value	HR (95% CI)	*p* value	*p* value	HR (95% CI)	*p* value
Age	<0.001			<0.001		
<=50		Reference			Reference	0.220
51–60		1.33 (1.21–1.46)	<0.001		1.05 (0.93–1.20)	0.396
61–70		2.01 (1.83–2.19)	<0.001		1.08 (0.95–1.24)	<0.001
71–80		3.57 (3.26–3.91)	<0.001		1.38 (1.21–1.58)	<0.001
>80		7.23 (6.57–7.95)	<0.001		2.14 (1.86–2.46)	<0.001
Race	<0.001			<0.001		
White		Reference			Reference	
Black		1,0.23 (1.20–1.27)	<0.001		1.12 (1.05–1.18)	<0.001
Other		0.78 (0.74–0.83)	<0.001		0.76 (0.69–0.83)	<0.001
Marital status	<0.001			<0.001		
Married		Reference			Reference	
Divorced		1.50 (1.45–1.56)	<0.001		1.32 (1.23–1.42)	<0.001
Separated		1.40 (1.27–1.55)	<0.001		1.26 (1.05–1.51)	0.013
Widowed		1.37 (1.32–1.43)	<0.001		1.31 (1.21–1.42)	<0.001
Single		1.32 (1.27–1.37)	<0.001		1.27 (1.19–1.35)	<0.001
T (tumor invasion)	<0.001			<0.001		
T1		Reference			Reference	
T2		1.02 (0.99–1.05)	0.104		1.10 (1.04–1.17)	<0.001
T3		1.26 (1.20–1.32)	<0.001		1.53 (1.42–1.65)	<0.001
T4		1.63 (1.53–1.74)	<0.001		1.86 (1.72–2.02)	<0.001
*N* (regional lymph node)	<0.001			<0.001		
No		Reference			Reference	
Yes		1.22 (1.15–1.29)	<0.001		1.24 (1.15–1.32)	<0.001
M (metastasis)	<0.001			<0.001		
No		Reference			Reference	
Yes		3.04 (2.90–3.19)	<0.001		5.54 (5.18–5.92)	<0.001
Surgery	<0.001			<0.001		
No		Reference			Reference	
Radical prostatectomy		0.35 (0.33–0.36)	<0.001		0.32 (0.30–0.35)	<0.001
Other surgical methods		0.81 (0.78–0.84)	<0.001		0.76 (0.71–0.81)	<0.001
Radiation	<0.001			0.037		
Yes		Reference			Reference	
No		1.35 (1.31–1.39)	<0.001		1.72 (1.38–2.17)	<0.001
None/Unknown		1.33 (1.17–1.52)	<0.001		1.40 (1.33–1.48)	<0.001
Chemotherapy	<0.001			<0.001		
None/Unknown		Reference			Reference	
Yes		1.56 (1.42–1.71)	<0.001		1.46 (1.30–1.63)	<0.001
Gleason score	<0.001			<0.001		
<=6		Reference			Reference	
3 + 4		1.24 (1.20–1.27)	<0.001		1.90 (1.75–2.06)	<0.001
4 + 3		1.40 (1.35–1.46)	<0.001		3.29 (3.02–3.59)	<0.001
8 (3 + 5/4 + 4/5 + 3)		1.65 (1.59–1.72)	<0.001		5.15 (4.75–5.58)	<0.001
9–10 (4 + 5 /5 + 4/5 + 5)		2.56 (2.46–2.67)	<0.001		10.23 (9.47–11.06)	<0.001
PSA value	<0.001			<0.001		
<=40		Reference			Reference	
41–100		1.12 (1.07–1.17)	<0.001		1.02 (0.93–1.12)	0.701
>100		1.59 (1.52–1.66)	<0.001		1.81 (1.65–1.99)	<0.001

Abbreviations:CI, confidence interval; HR, hazard ratio.

### 3.3 Prognostic nomogram for survival

**FIGURE 2:**
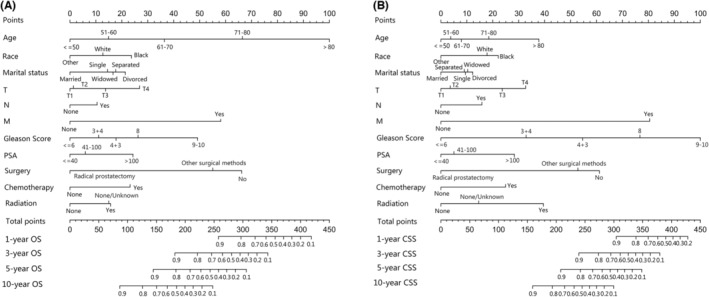
Nomograms for predicting 1‐, 3‐, 5‐, and 10‐year overall survival (A) and cancer‐specific survival (B) rate of prostate cancer.

**FIGURE 3:**
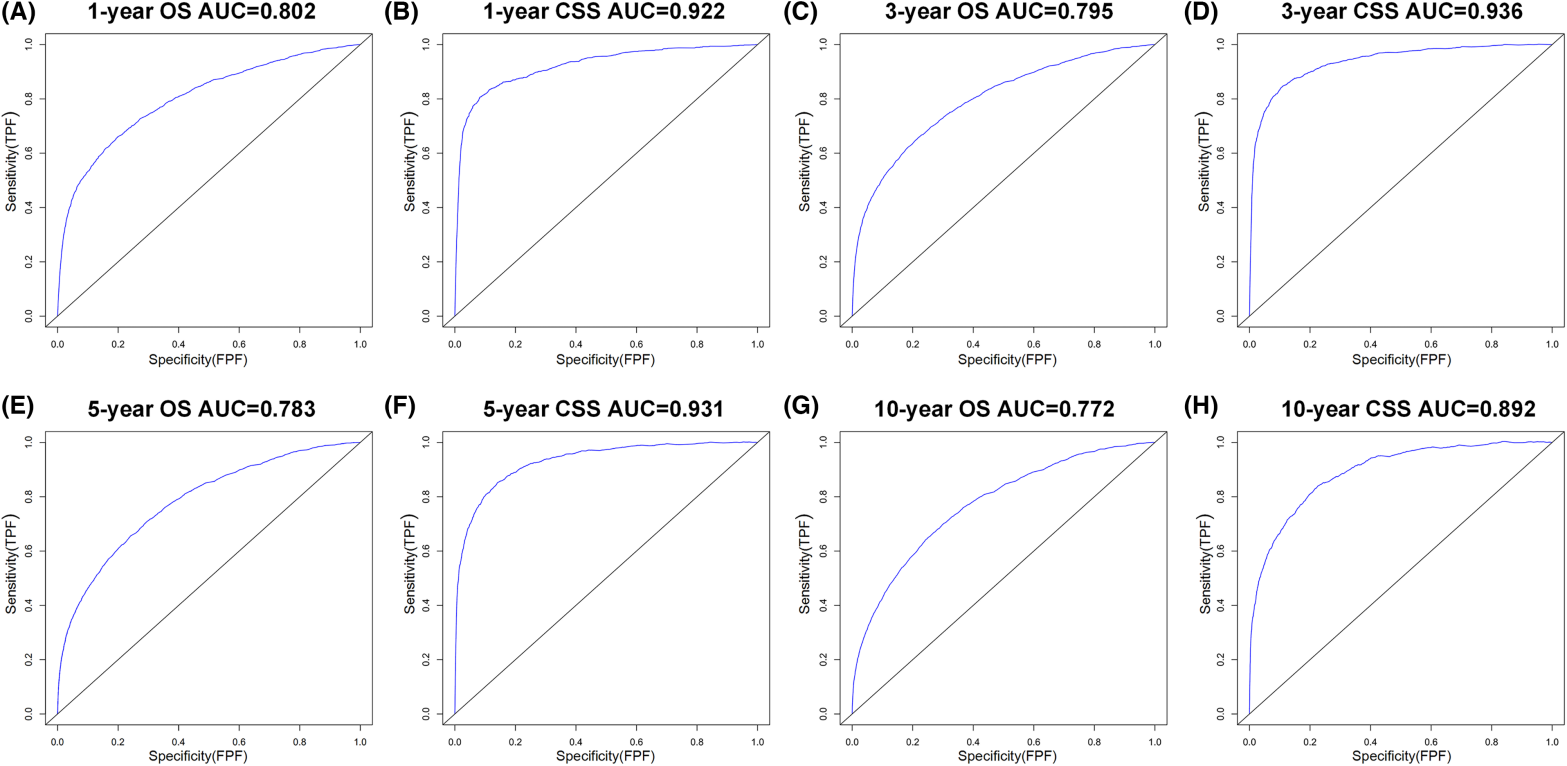
Receiver operating characteristic (ROC) curve to test the ability of independent prognostic factors predicting 1‐, 3‐, 5‐, and 10‐year overall survival (OS) and cancer‐specific survival (CSS) in prostate patients: (A) 1‐year OS; (B) 1‐year CSS; (C) 3‐year OS; (D) 3‐year CSS; (E) 5‐year OS; (F) 5‐year CSS. (G) 10‐year OS; (H) 10‐year CSS.

**FIGURE 4:**
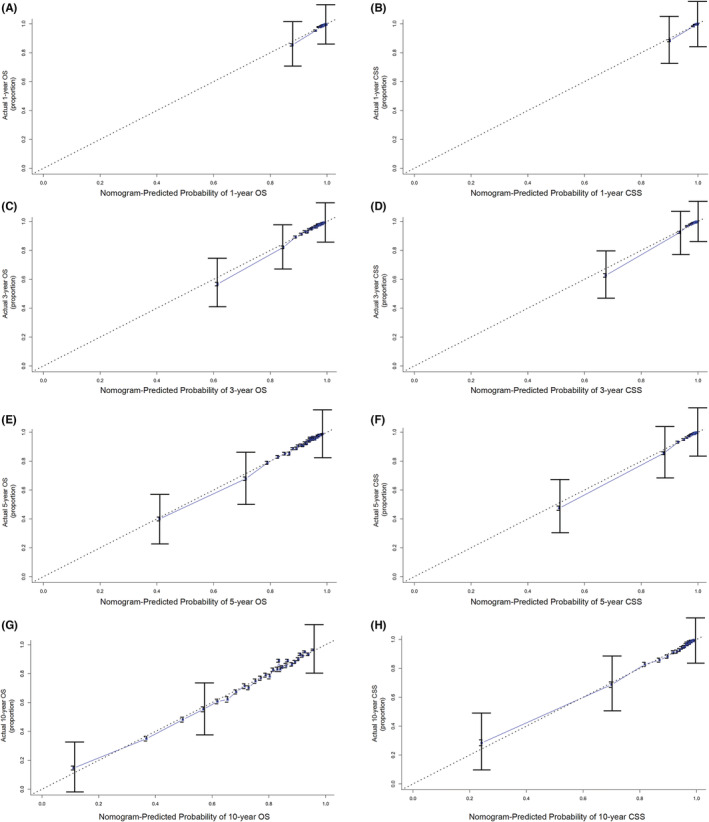
Calibration curves for 1‐, 3‐, 5‐, and 10‐year overall survival (OS) and cancer‐specific survival (CSS) for prostate cancer patients in the training cohort: (A) 1‐year OS; (B) 1‐year CSS; (C) 3‐year OS; (D) 3‐year CSS; (E) 5‐year OS; (F) 5‐year CSS. (G) 10‐year OS; (H) 10‐year CSS.

**FIGURE 5:**
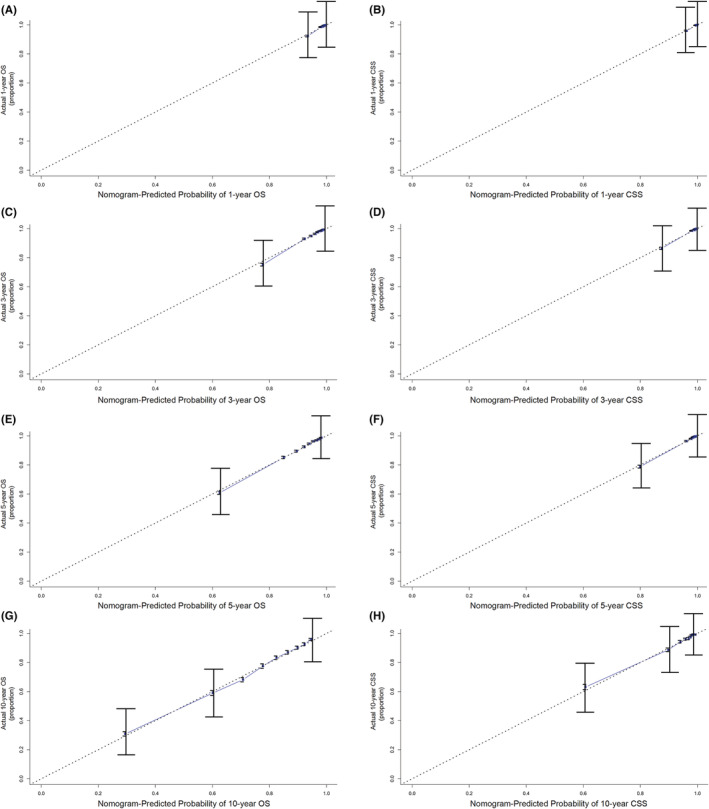
Calibration curves for 1‐, 3‐, 5‐, and 10‐year overall survival (OS) and cancer‐specific survival (CSS) for prostate cancer patients in the validation cohort: (A) 1‐year OS; (B) 1‐year CSS; (C) 3‐year OS; (D) 3‐year CSS; (E) 5‐year OS; (F) 5‐year CSS. (G) 10‐year OS; (H) 10‐year CSS.

**FIGURE 6:**
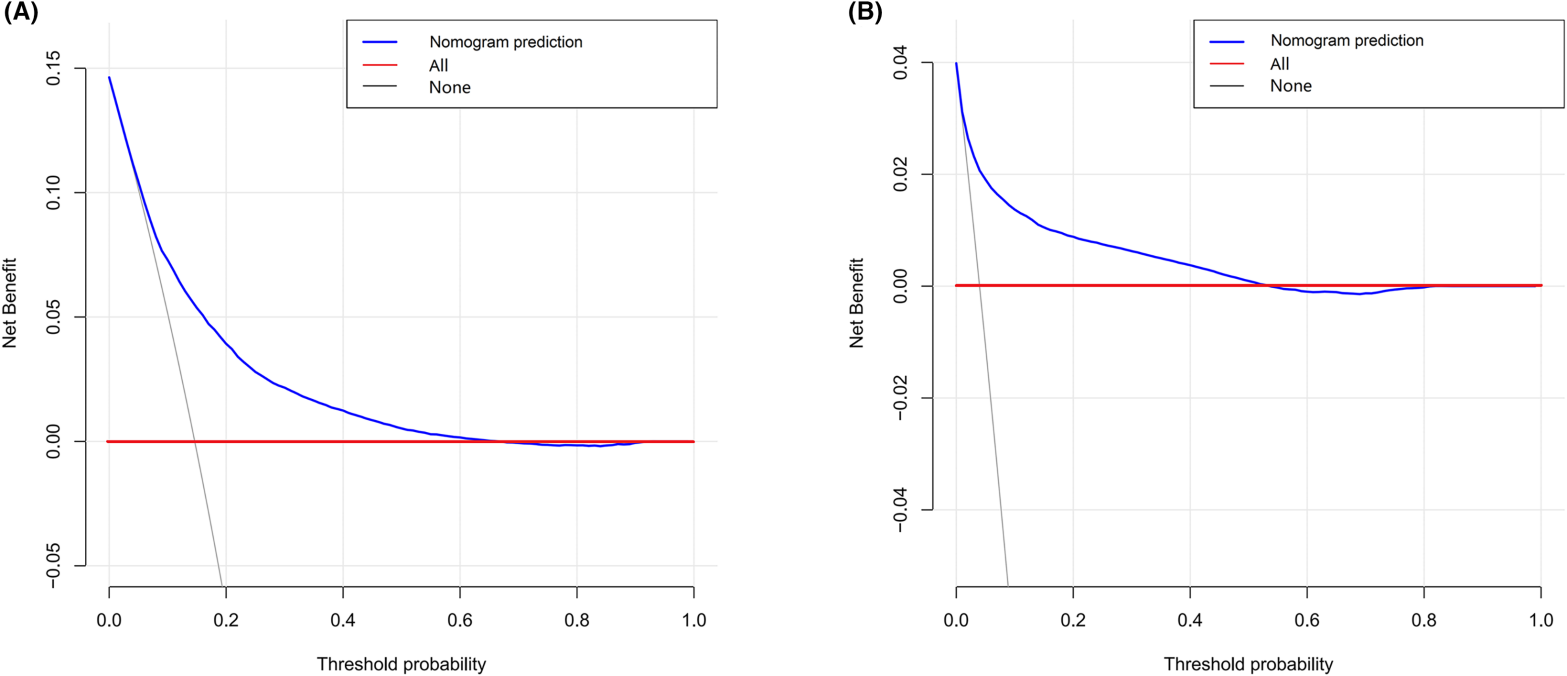
DCA curves to evaluate the practicability of the nomogram models for decision‐making. (A) DCA curves for OS in the training cohort; (B) DCA curves for CSS in the training cohort. The gray line indicates patients who died during the follow‐up period, and the red line indicates that no patients died. The solid blue line represents the net benefit of the nomogram prediction.

The independent prognostic factors, race, age at diagnosis, marital status, TNM stage, surgery method, radiotherapy, chemotherapy, prostate‐specific antigen (PSA) value and Gleason score were combined to establish nomograms to predict 1‐, 3‐, 5‐, and 10‐year OS and CSS rates in patients with PCa (Figure 2). The OS and CSS rate of PCa patients can be estimated by adding the scores corresponding to each variable on the nomogram. We established ROC curves to further evaluate the ability of independent prognostic factors to predict 1‐, 3‐, 5‐, and 10‐year OS and CSS. As expected, results also indicated the good ability to predict the survival of PCa patients (Figure 3), as indicated by the AUC generated from the model (for 1‐, 3‐, 5‐, and 10‐year OS, AUC = 0.802, 0.795, 0.783 and 0.772, respectively; and for 1‐, 3‐, 5‐, and 10‐year CSS, AUC = 0.922, 0.936, 0.931 and 0.892, respectively). The accuracy of the nomogram model for survival prediction was tested and externally validated by C‐index value and correction curve. The advantageous C‐index values (for training validation: OS C‐index =0.776, 95%CI = 0.774–0.778; CSS C‐index = 0.889, 95%CI = 0.885–0.893; and for external validation: OS C‐index =0.759, 95%CI = 0.755–0.763; CSS C‐index = 0.875, 95%CI = 0.869–0.881) and the approximation between prediction and observation in correction curves indicated that the nomograms models had good accuracy in discrimination of patients' survival (Figure 4 and Figure 5). Internally validation using the DCA curves demonstrated that the prognostic nomogram had good practicability for clinical decision‐making (Figure 6).

### 3.4 Establishment of prognostic scoring system

**FIGURE 7:**
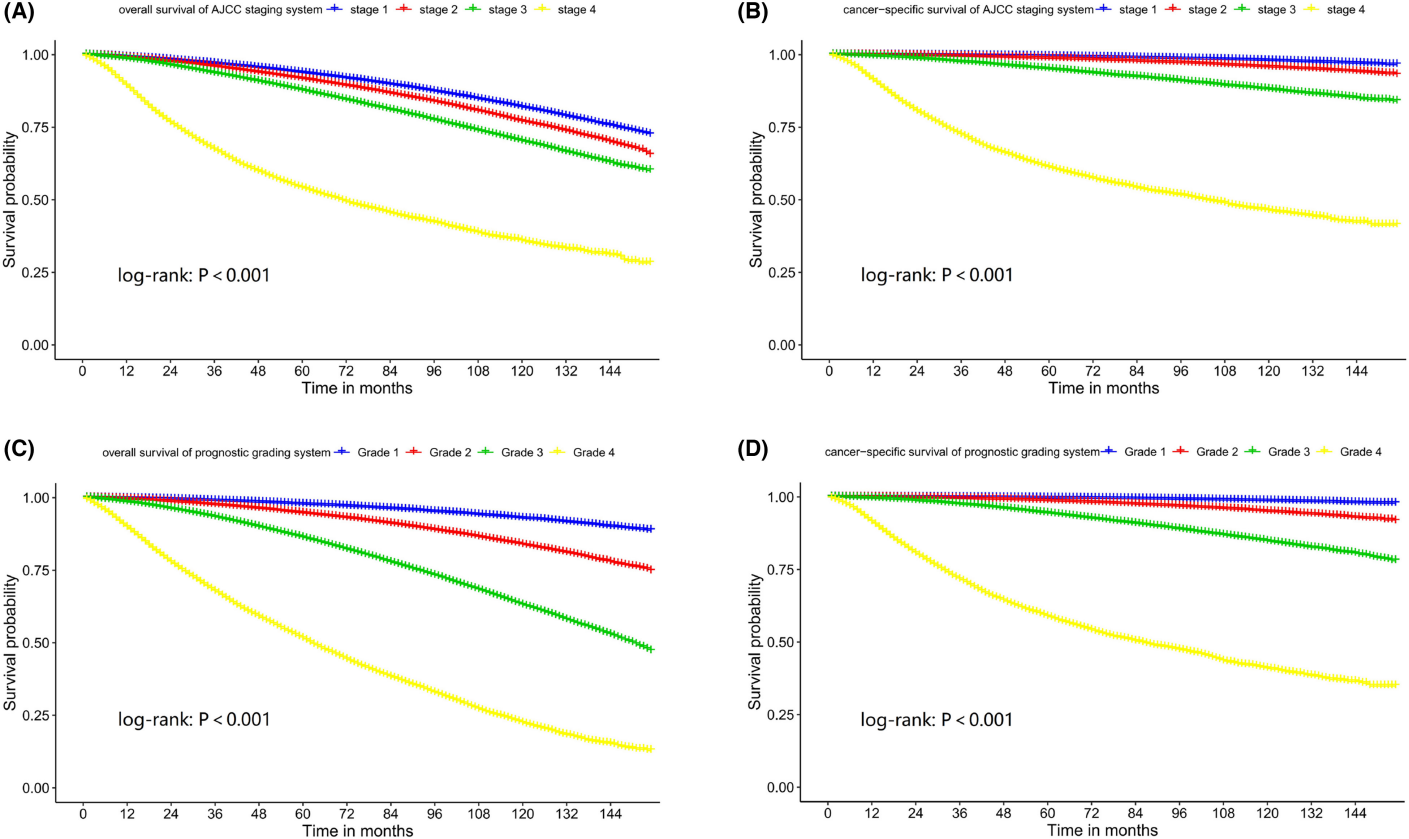
The Kaplan–Meier curves comparison of overall survival (OS) and cancer‐specific survival (CSS) between prostate cancer patients divided with AJCC staging system and divided with the prognostic grading system: (A) OS of AJCC staging system; (B) CSS of AJCC staging system; (C) OS of prognostic grading system; (D) OS of prognostic grading system.

We included the independent prognostic factors in Cox models and resulted in HRs presented in Table 2. We calculated prognostic scores for OS and CSS according to the coefficient in the Cox models (Table 3 and Table 4). The prognostic scoring system has a total score range 0–100, and a higher score indicated a worse prognosis of PCa patients. We divided the prognosis of PCa patients into four grades based on the prognostic scoring system: 0–25 points (grade 1), 26–35 points (grade 2), 36–50 points (grade 3), and 51–100 points (grade 4). Kaplan–Meier curves were used to compare the OS and CSS of PCa patients in different grades or stages using the prognostic grading system and AJCC staging system (Figure 7). The proportion of patients, OS and CSS rates for each AJCC stage and prognostic grade were presented in Table 5. We used AIC to evaluate the suitability of survival models for both grading systems, and found that the use of prognostic grading system was superior to the AJCC staging system in assessing patient outcomes, especially for OS (AIC values: prognostic grading system OS = 9.93 × 10^5^, CSS = 2.55 × 10^5^; AJCC staging system OS = 10.24 × 10^5^, CSS = 2.64 × 10^5^).

**TABLE 3:** Scores of independent prognostic factors in the prognostic scoring system for all‐cause survival

Patient characteristics	All‐cause death points	Patient characteristics	All‐cause death points	Patient characteristics	All‐cause death points
Age		T stage		Surgery	
<=50	0	T1	0	No	16
51–60	4	T2	0	RP	0
61–70	9	T3	4	Other	13
71–80	16	T4	6	Radiation	
>80	23	*N* stage		Yes	0
Race		No	0	No	4
White	4	Yes	4	None/Unknown	4
Black	6	M stage		Chemotherapy	
Other	0	No	0	Yes	5
Marital status		Yes	13	None/Unknown	0
Married	0	Gleason score		PSA value	
Divorced	5	<=6	0	<=40	0
Separated	4	3 + 4	3	41–100	2
Widowed	4	4 + 3	4	>100	6
Single	4	8 (3 + 5/4 + 4/5 + 3)	6		
		9–10 (4 + 5/5 + 4/5 + 5)	12		

*Note*: The prognostic scoring system has a total score of 100, with a higher score indicating a worse prognosis of prostate cancer patients. We divided the prognosis of prostate cancer patients into four grades: 0–25 points (grade 1), 26–35 points (grade 2), 36–50 points (grade 3) and 51–100 points (grade 4).

**TABLE 4:** Scores of independent prognostic factors in the prognostic scoring system for cancer‐specific survival

Patient characteristics	All‐cause death points	Patient characteristics	All‐cause death points	Patient characteristics	All‐cause death points
Age		T stage		Surgery	
<=50	0	T1	0	No	14
51–60	1	T2	1	RP	0
61–70	2	T3	5	Other	12
71–80	4	T4	7	Radiation	
>80	8	*N* stage		Yes	0
Race		No	0	No	7
White	4	Yes	3	None/Unknown	4
Black	5	M stage		Chemotherapy	
Other	0	No	0	Yes	5
Marital Status		Yes	18	None/Unknown	0
Married	0	Gleason score		PSA value	
Divorced	3	<=6	0	<=40	0
Separated	2	3 + 4	6	41–100	2
Widowed	2	4 + 3	12	>100	6
Single	2	8 (3 + 5/4 + 4/5 + 3)	17		
		9–10 (4 + 5/5 + 4/5 + 5)	24		

*Note*: The prognostic scoring system has a total score of 100, with a higher score indicating a worse prognosis of prostate cancer patients. We divided the prognosis of prostate cancer patients into four grades: 0–25 points (grade 1), 26–35 points (grade 2), 36–50 points (grade 3) and 51–100 points (grade 4).

**TABLE 5:** The comparison of overall survival (OS) and cancer‐specific survival (CSS) between prostate cancer patients divided with AJCC staging system and divided with the prognostic grading system

Stage (Grade)	Survival time	AJCC staging system			Prognostic grading system			
			OS	CSS	OS		CSS	
		Proportion (%)	Rate (%)	Rate (%)	Proportion (%)	Rate (%)	Proportion (%)	Rate (%)
I	3‐year	41.9	96.9	99.7	32.6	99.0	49.1	99.8
	5‐year		93.7	99.4		97.8		99.6
	10‐year		81.8	97.9		92.9		98.7
II	3‐year	32.6	95.9	99.4	31.2	97.5	30.7	99.4
	5‐year		91.7	98.7		94.6		98.7
	10‐year		77.0	95.7		83.8		95.0
III	1‐year	21.2	98.5	99.5	29.1	98.5	15.1	99.5
	3‐year		93.6	97.5		93.3		97.5
	5‐year		87.7	95.0		86.2		94.4
	10‐year		70.2	88.1		63.0		84.7
IV	1‐year	4.3	89.3	91.2	7.1	89.7	5.1	91.4
	3‐year		67.3	72.4		67.6		71.6
	5‐year		54.1	61.1		51.5		58.7
	10‐year		35.8	46.3		22.3		40.9

## 4 DISCUSSION

At present, medical data mining is increasingly applied to clinical practice.^14^ Clinical big data plays an important role in establishing prognostic models, assessing risk factors, diagnosis and treatment of diseases, which benefit patients greatly.^15^, ^16^ Prediction of cancer survival is an important part of oncology. The main advantage of nomograms is the individualized risk assessment based on the different information of patients, which benefits clinical practice in many aspects such as disease prediction, tumor recurrence, survival assessment and adjuvant therapy^.^ ^17^, ^18^, ^19^, ^20^ In this population‐based study, we developed prognostic nomograms based on clinical characteristics and treatment of PCa patients. Statistical methods such as C‐index, ROC curve, DCA and Calibration curves were used to evaluate the prediction accuracy of the Nomogram. The C‐index and AUC value can be good tests for the ability of nomogram prediction, and the value close to 1 means higher accuracy.^17^, ^21^ DCA is used to evaluate the practicability of prediction model for decision‐making.^22^ The similarity between the predicted curve and the observed curve in the calibration curves can directly demonstrate the prediction ability of nomogram.^17^ Internal and external validation demonstrated that the proposed nomograms have high accuracy and excellent discrimination ability in predicting 1‐, 3‐, 5‐, 10‐year OS and CSS rate for PCa patients. We further established the prognostic scoring system and verified that it was more effective than the traditional AJCC staging system in predicting the prognosis of PCa patients, especially for OS. Effective individualized survival prediction tools may contribute to clinical practice in selecting effective treatment options and intensity for PCa patients.

In this study, age at diagnosis, race, marital status, TNM stage, surgery method, radiotherapy, chemotherapy, PSA value and Gleason score were identified as independent prognostic factors that were included for the development of prognostic nomograms. Many studies have identified race as an independent risk factor for the survival of PCa patients, and black patients have worse survival than white men, which is due, in part, to the relatively low economic and medical environment.^23^, ^24^ Previous studies had demonstrated that marital status was able to affect survival in patients with many types of cancer, such as prostate, breast and lung cancer.^25^ Married cancer patients tend to have better survival than those unmarried (divorced/widowed/separated), and the associated benefits were more significant in males than females.^25^ A population‐based study had reported that in all tumor stages of PCa patients, the OS and CSS of married men are higher than that of unmarried men, which may be related to patients' physical and mental health and treatment compliance.^26^ In our study, the effects of race and marital status on OS and CSS of PCa patients are similar to previous reports.

The clinical information of PC patients in this study, including age, PSA, TNM stage and Gleason value, was based on the registration of patients at the time of initial diagnosis. However, the PSA value in patients with PCa is a dynamic indicator, and the SEER database does not provide PSA monitoring information for patients who underwent subsequent treatment, such as radical prostatectomy, radiotherapy, and androgen deprivation therapy. Although PSA is controversial as a routine screening test for PCa due to its diversity of effects, it is widely accepted as an indicator for treatment effectiveness and biochemical recurrence of PCa.^27^, ^28^, ^29^ Biochemical recurrence time and PSA increase speed were important factors affecting survival of PCa patients after treatment.^30^, ^31^, ^32^ In our survival prognosis model, PSA value was not significantly associated with OS and CSS, but we could not provide the results regarding the dynamic effect of PSA value on patients' survival.

We purposed a PCa prognostic scoring system using the clinicopathological information that was associated with patients' survival. Age was the highest risk factor for OS of PCa, which scored patients 0 to 23. Gleason score was weighed 0–24 score to patients, which was the highest risk factor for CSS. The classification of the AJCC system for PCa was based on Gleason score and tumor TNM stage. In our prognostic scoring system, we found that Gleason score and TNM staging in combination weighed different scores for OS and CSS (e.g., 0–35 points for OS and 0–52 points for CSS), which is the main reason for the large difference for OS but less difference for CSS in the Kaplan–Meier curves in the comparison of our prognostic scoring system versus AJCC staging system.

The advantage of establishing prognostic assessment tools is that it can provide an intuitive initial survival expectation, based on which clinicians and patients can jointly determine treatment options. However, predictive tools are not a substitute for clinical judgment, and clinicians need to make trade‐offs based on individual differences such as the severity of comorbidities and physical conditions. Although this is not the first nomogram proposed to assess survival in PCa patients, previously proposed prognostic tools were mostly limited to localized PCa.^33^, ^34^ Because our prognostic tool considered comprehensive clinical factors and included PCa patients with advanced stage, it may provide new clues for possible clinical translation.

However, there are some limitations in our study. First, the PSA growth rate and biochemical recurrence time of PCa patients are important indicators for survival,^35^ but the missing information in SEER database may reduce the accuracy of our prediction. The second, the health status and concomitant diseases of PCa patients were not included in our study, which may introduce bias into our results. Last, data included this study was retrospectively collection, and more prospective studies are needed to confirm our results.

## 5 CONCLUSIONS

Based on a large data set of PCa patients, we developed and validated prognostic nomograms and demonstrated their favorable ability in predicting 1‐, 3‐, 5‐, and 10‐year OS and CSS for PCa patients. We further proposed the prognostic scoring system for the first time to more intuitively present the influence of clinical pathological factors on OS and CSS of PCa. The proposed survival model of PCa can help clinicians select individualized and effective treatment.

## AUTHOR'S CONTRIBUTION

Yuanyuan Chang and Lei Cheng designed and reviewed this research. Yuan Zhou and Changming Lin completed statistical analysis and the draft manuscript. Lian Zhu and Rentao Zhang performed validation and processed the charts.

## CONFLICT OF INTEREST

The authors declare that the research was conducted in the absence of commercial or financial relationships that could be construed as a potential.

## ETHICS APPROVAL AND CONSENT TO PARTICIPATE

The data for this study are publicly available. Therefore, informed consent of patients and permission of the ethics committee were not required for this study.

## Supporting information


Table S1

## Data Availability

The publicly datasets analyzed in the present study can be found here: https://seer.cancer.gov/data/.
