# Supplementary material for: Nomograms and scoring system for forecasting overall and cancer‐specific survival of patients with prostate cancer
Source: Cancer Med. 2022 Aug 22;12(3):2600–13. doi: 10.1002/cam4.5137 (PMC9939188; doi:10.1002/cam4.5137)
Supplement: Supplementary file 1 — Table S1 [file CAM4-12-2600-s001.docx]

| **Supplementary Table 1:** The distribution of invasion factors in prostate cancer. | | | | | |
| --- | --- | --- | --- | --- | --- |
| **Patients**  **Characteristics** | **Gleason Score (Number of patients)** | | | | |
|  | **<=6** | **3+4** | **4+3** | **8** | **9-10** |
| Number of patients | 124,236 | 81,209 | 33,650 | 24,280 | 19,877 |
| T stage (%) |  |  |  |  |  |
| T1 | 55.2 | 35.1 | 37.0 | 39.6 | 35.6 |
| T2 | 40.7 | 49.0 | 38.7 | 33.4 | 24.3 |
| T3 | 3.6 | 14.7 | 22.2 | 23.2 | 30.1 |
| T4 | 0.5 | 1.2 | 2.1 | 3.8 | 10.0 |
| N stage (%) |  |  |  |  |  |
| Yes | 0.2 | 1.2 | 3.6 | 7.2 | 16.9 |
| No | 99.8 | 98.8 | 96.4 | 92.8 | 83.1 |
| M stage (%) |  |  |  |  |  |
| Yes | 0.2 | 0.6 | 1.9 | 6.8 | 17.9 |
| No | 99.8 | 99.4 | 98.1 | 93.2 | 82.1 |
| PSA value (ng/ml) |  |  |  |  |  |
| Median | 50 | 63 | 74 | 90 | 121 |
| IQR | 44-80 | 47-95 | 52-124 | 57-182 | 67-353 |
| **Note:** Abbreviations: IQR= interquartile range. | | | | | |
